# Delirium in the intensive care unit and its importance in the post-operative context: A review

**DOI:** 10.3389/fmed.2023.1071854

**Published:** 2023-03-30

**Authors:** Danielle Ní Chróinín, Evan Alexandrou, Steven A. Frost

**Affiliations:** ^1^Liverpool Hospital, Liverpool, NSW, Australia; ^2^South Western Sydney Clinical School, UNSW Sydney, Liverpool, NSW, Australia; ^3^Centre for Applied Nursing Research, School of Nursing and Midwifery, Western Sydney University and Ingham Institute of Applied Medical Research, Liverpool, NSW, Australia; ^4^School of Nursing, Faculty of Science, Medicine and Health, University of Wollongong, Wollongong, NSW, Australia; ^5^SWS Nursing and Midwifery Research Alliance, Ingham Institute of Applied Medical Research, Liverpool, NSW, Australia

**Keywords:** delirium, post-operative, intensive care unit, nursing, multidisciplinary, cognitive impairment

## Abstract

The burden of delirium in the intensive care setting is a global priority. Delirium affects up to 80% of patients in intensive care units; an episode of delirium is often distressing to patients and their families, and delirium in patients within, or outside of, the intensive care unit (ICU) setting is associated with poor outcomes. In the short term, such poor outcomes include longer stay in intensive care, longer hospital stay, increased risk of other hospital-acquired complications, and increased risk of hospital mortality. Longer term sequelae include cognitive impairment and functional dependency. While medical category of admission may be a risk factor for poor outcomes in critical care populations, outcomes for surgical ICU admissions are also poor, with dependency at hospital discharge exceeding 30% and increased risk of in-hospital mortality, particularly in vulnerable groups, with high-risk procedures, and resource-scarce settings. A practical approach to delirium prevention and management in the ICU setting is likely to require a multi-faceted approach. Given the good evidence for the prevention of delirium among older post-operative outside of the intensive care setting, simple non-pharmacological interventions should be effective among older adults post-operatively who are cared for in the intensive care setting. In response to this, the future ICU environment will have a range of organizational and distinct environmental characteristics that are directly targeted at preventing delirium.

## Introduction

The burden of delirium in the intensive care setting is a global priority (1, 2). Delirium is an acute neurocognitive disorder that is characterized by a fluctuating level of consciousness with impaired attention and cognition (3). Delirium affects up to 80% of patients in intensive care units (4). An episode of delirium is often distressing to patients and their families, and in patients within, or outside of, the intensive care unit (ICU) setting, and it is associated with poor outcomes, in the short term, which includes longer stay in intensive care, longer hospital stay, and increased risk of hospital mortality in patients (5–11). Longer term sequelae include cognitive impairment and dependency in activities of daily living (6, 9, 12–16). In the Australian healthcare setting, it has been estimated that an episode of delirium increases hospital stay by, on average, 2.7 days (17), and in the ICU-based Deli I study, patients experiencing an acute episode of delirium stayed, on average, an extra 6 days longer in hospital (18).

## Delirium in the intensive care setting

Each year, there are approximately 175,000 admissions to Australian adult intensive care units (ICUs); this number has been increasing by 6% each year since 2011 (19). While there is considerable variability in the intensive care unit admissions depending on geographic location (20), intensive care unit beds and usage appear to be increasing (20). The majority of patients admitted to intensive care will survive ICU (19, 20); however, as many as one in five patients will experience an acute episode of delirium (21), and being older and frail increases the risk (2, 11, 22–24). The direct healthcare costs associated with delirium and longer hospital stay alone would be approximately $255 million annually in the Australian intensive care setting, excluding the cost due to the loss of healthy life, which has been estimated to be double that of direct healthcare costs (17, 25).

While the medical category of admission may be a risk factor for poor outcomes (26, 27) in critical care populations, outcomes for surgical ICU admissions are not particularly optimistic, with dependency at hospital discharge exceeding 30% (28) and average in-hospital mortality in the order of approximately 2.5–5% but exponentially higher in older patients or those undergoing high-risk procedures (28–31). Thirty-day mortality among non-cardiac surgical patients reaches almost 40% (32); even higher mortality rates have been observed in resource-limited settings (33). A recent study indicated that 28% of 350,000 admissions across 238 ICUs in the United States represented a primary surgical diagnosis (28). While encouraging trends were noted in terms of mortality and length of stay for some surgical cohorts, functional decline appeared to be increasing over time (28). Factors such as delirium, prolonged immobilization, and mechanical ventilation may all contribute to functional decline and other poor outcomes in surgical and general ICU populations, exacerbated by underlying risk factors such as age, frailty, comorbidity, and cognitive impairment (28, 34–36). Although not specific to those requiring intensive care admission, post-operative delirium is reported in upward of 65% of patients (37, 38). Identification of those who have the highest risk may facilitate the implementation of targeted interventions (39). The risk for the development of post-operative delirium may be conceptualized as relating to pre-operative (baseline) factors, intra-operative factors related to the surgery and anesthetic, and post-operative factors (38). A recent study highlighted the potential to predict delirium in older (aged ≥70 years) surgical patients undergoing elective cardiovascular, orthopedic, or general surgery (40), with surgery type, multimorbidity, renal failure, polypharmacy, ASA, cut-to-suture time, and cognitive assessment allowing an ability to predict delirium with an AUC of 0.8 (40). This information is helpful not only just in planning care but also in discussing risk with patients and families and managing expectations. Furthermore, embedding assessment in formal multi-faceted structures such as comprehensive geriatric assessment (CGA) may reduce post-operative delirium in older patients such as those undergoing vascular or hip fracture surgery (41, 42).

The good news is that high-quality evidence suggests that at least 30% of episodes of hospital-acquired delirium are preventable, including, for example, in post-operative hip fracture cohorts (3, 43, 44). Multi-component, multidisciplinary interventions have been shown to reduce the incidence of delirium, in general wards, post-operative, and aged care settings (3, 45–48). However, evidence for the effectiveness of interventions to reduce the burden of delirium in the intensive care has been inconclusive (49–54), and none of these intensive care studies focused purely on post-operative populations. Gaps are in part attributable to a lack of focus on the effective implementation and dissemination of evidence into practice (55–57). There is a lack of good evidence supporting the use of pharmacological interventions to prevent delirium in the intensive care setting. A recent Cochrane systematic review (45) concluded that “the effects of other pharmacological, sedation, environmental, and preventive nursing interventions is unclear and warrants further investigation,” while a meta-analysis of bundle interventions likewise failed to show an association with delirium prevalence or duration (58). Nonetheless, previous trials, systematic reviews, and meta-analyses have shown promise in terms of the effectiveness of non-pharmacological interventions to reduce the burden of delirium in the hospital and critical care settings (43–47, 52).

A recent review of pharmacological therapy in the ICU highlighted the significant limitations of existing trials, with heterogeneity in terms of agents used, primary outcome measures, timing of treatment, and delirium diagnosis (59). Among the available pharmacological agents, dexmedetomidine has some evidence supporting its benefit in reducing post-operative delirium in older patients undergoing elective non-cardiac surgery (60). A slightly more recent meta-analysis of 14 melatonin/ramelteon studies suggested that these formulations might significantly reduce delirium in surgical (49% risk reduction) and ICU (34%) patient groups (61), but optimum duration, dosing, and formulation are yet to be identified.

In addition to delirium prevention, early recognition of delirium is key. Improving detection through the use of screening tools (3, 62, 63) may facilitate improved diagnosis, which can in turn trigger prompts to guide investigation and management (3, 64, 65). Simple screening tools may in fact be utilized to assist in the diagnosis of delirium in the intensive care setting. The Confusion Assessment Method (CAM) and its ICU version have been validated as a reliable (kappa = 0.96; 95% CI 0.91–0.99) and valid (sensitivity 0.81–0.82 and specificity 0.99) tool to diagnose delirium in the intensive care setting (66–68). Hypoactive delirium, which is common in older patients, is associated with a poorer prognosis than the hyperactive form (3) but is more likely to be under-recognized (69), highlighting the need to maintain an appropriate index of suspicion in older patients. While DSM-V criteria for the diagnosis of delirium no longer explicitly refer to the level of arousal for the diagnosis of delirium, the level of arousal is fundamental to the assessment of attention and cognition and should be included in the assessment of the potentially delirious patient (70). The issue of coma is also pertinent to the ICU setting, and it is worth noting that a diagnosis of delirium is precluded in patients with a severely reduced level of arousal such as coma (71).

Thus, a practical approach to delirium prevention and management in the ICU setting is likely to require a multi-faceted approach. Some examples of non-pharmacological interventions to reduce the risk of delirium are presented in the Table 1. Environmental factors may also be a focus of risk-reduction strategies, with design modifications potentially targeting sound and light, floor planning, and room arrangement, aiming to reduce stressors and positively influence the patient experience (4). Harnessing the expertise and manpower of family members, to assist with aspects of care such as orientation and memory cueing, cognitive stimulation, and sensory checks, may also be feasible and acceptable (72).

**Table 1:** Non-pharmacological interventions reduce the risk of delirium.

Component	Intervention
Cognitive impairment.	Establish a baseline using the validated CAM, CAM-ICU assessment tool and use orientation techniques (14, 53, 74).
	All patients will be re-orientated to time/place/people/event such as reason for hospitalization, at regular intervals (53, 75–77)
Sensory functions.	Optimise sensory function for vision and hearing by ensuring glasses and hearing aids are available and appropriately used when patient is awake. Families will be reminded to have these items available, and nurses will ensure their appropriate use (78, 79)
	Use appropriate communication technique (verbal/written/pictures) to compensate sensory loss and overcome language barriers (76, 79, 80)
Environmental interventions	Provide visible clock, calendar and schedule for each patient (74–77, 80–85)
	Provide sleep management (night light, foot massage, back massage) (74, 76, 77, 84)
	Provide comfortable physical environment (reducing noise, persistent nursing, the limited movement to other beds, beds areas, and allow to bring home favorites) (16, 78, 79, 81, 84–90)
	Remove physical restraints as soon as feasible, contingent to patient’s safety (16, 87, 90)
	Arrange familiar people to visit and encourage family visitors to stay longer and frequently when possible, especially for patients with non-English speaking background and during planned sedation weaning (76, 80, 91)
Early therapeutic interventions	Encourage early mobilization and plan mobility schedule (74, 92)
	Provide appropriate nutrition; keep fluid and electrolyte balance (67)
	Assessing and addressing pain management effectively and early (87, 93)
	Careful use of sleeping pills, anticholinergics and opiates (87)
	Avoid hypoxia.
	Early detection and management of infection.
	Removal of unnecessary catheters (87, 94)
	Routinely screen alcohol history and commence Alcohol Withdrawal Assessment where appropriate (87)

## Implications for clinical practice

Good quality evidence suggests that at least 30% of episodes of delirium among older adults admitted to the hospital are preventable, with interventions being delivered by an interdisciplinary team of nursing, medical, and allied health clinicians (3). There is consistent evidence that these multi-component interventions are effective in preventing delirium, in general wards and aged care settings (43, 45). However, evidence for the effectiveness of interventions to reduce the burden of delirium in the intensive care has been inconclusive. While and small single-site, non-pharmacological multi-component interventional studies have shown promising results (45), larger studies, often among patients at high risk, have not shown a clear benefit (43, 49). In particular, older cardiothoracic surgery patient appears to be resistant to intervention in the ICU, even when other similar-aged surgical patients can have the risk of reduced post-operative delirium (43). Importantly, several significant organizational and design changes to the intensive care setting have been proposed, as “the future of intensive care: delirium should no longer be an issue” (73).

## Conclusion

Given the good evidence for the prevention of delirium among older post-operative outside of the intensive care setting, simple non-pharmacological interventions should be effective among older adults post-operatively cared for in the intensive care setting. In response to this, the future ICU environment will have a range of organizational and distinct environmental characteristics that are directly targeted at preventing delirium.

## Author contributions

DN, EA, and SF were responsible for drafting, editing, and finalization of the manuscript. All authors agreed to be accountable for the content of the study.

## Conflict of interest

The authors declare that the research was conducted in the absence of any commercial or financial relationships that could be construed as a potential conflict of interest.

## Publisher’s note

All claims expressed in this article are solely those of the authors and do not necessarily represent those of their affiliated organizations, or those of the publisher, the editors and the reviewers. Any product that may be evaluated in this article, or claim that may be made by its manufacturer, is not guaranteed or endorsed by the publisher.
